# A nonlinear mixed‐effects modeling approach for ecological data: Using temporal dynamics of vegetation moisture as an example

**DOI:** 10.1002/ece3.5543

**Published:** 2019-08-15

**Authors:** Facundo J. Oddi, Fernando E. Miguez, Luciana Ghermandi, Lucas O. Bianchi, Lucas A. Garibaldi

**Affiliations:** ^1^ IRNAD (UNRN, Sede Andina) and CONICET Río Negro Argentina; ^2^ Department of Agronomy Iowa State University Ames IA USA; ^3^ INIBIOMA (CONICET ‐ UNCo) Río Negro Argentina

**Keywords:** correlation structures, hierarchical modeling, nonlinearity, spatio‐temporal variability, time series

## Abstract

1. Increasingly, often ecologist collects data with nonlinear trends, heterogeneous variances, temporal correlation, and hierarchical structure. Nonlinear mixed‐effects models offer a flexible approach to such data, but the estimation and interpretation of these models present challenges, partly associated with the lack of worked examples in the ecological literature.
2. We illustrate the nonlinear mixed‐effects modeling approach using temporal dynamics of vegetation moisture with field data from northwestern Patagonia. This is a Mediterranean‐type climate region where modeling temporal changes in live fuel moisture content are conceptually relevant (ecological theory) and have practical implications (fire management). We used this approach to answer whether moisture dynamics varies among functional groups and aridity conditions, and compared it with other simpler statistical models. The modeling process is set out “step‐by‐step”: We start translating the ideas about the system dynamics to a statistical model, which is made increasingly complex in order to include different sources of variability and correlation structures. We provide guidelines and R scripts (including a new self‐starting function) that make data analyses reproducible. We also explain how to extract the parameter estimates from the R output.
3. Our modeling approach suggests moisture dynamic to vary between grasses and shrubs, and between grasses facing different aridity conditions. Compared to more classical models, the nonlinear mixed‐effects model showed greater goodness of fit and met statistical assumptions. While the mixed‐effects approach accounts for spatial nesting, temporal dependence, and variance heterogeneity; the nonlinear function allowed to model the seasonal pattern.
4. Parameters of the nonlinear mixed‐effects model reflected relevant ecological processes. From an applied perspective, the model could forecast the time when fuel moisture becomes critical to fire occurrence. Due to the lack of worked examples for nonlinear mixed‐effects models in the literature, our modeling approach could be useful to diverse ecologists dealing with complex data.

## Graphical abstract

Nonlinear mixed‐effects models offer a flexible approach to analyze complex data, but the estimation and interpretation of these models present challenges to ecologists, partly associated with the lack of developed examples in the literature. We illustrate the nonlinear mixed‐effects modeling approach using temporal dynamics of vegetation moisture with field data. We provide guidelines and R scripts that make data analyses reproducible and also explain how to extract the parameter estimates from the R output.

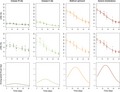

## 1 INTRODUCTION

Classic statistical approaches (e.g., linear regression or ANOVA) have assumptions that often are not met by ecological data, such as when variances change with predictors or responses are nonlinear (Bolker et al., 2013; Zuur, Ieno, Walker, Saveliev, & Smith, 2009). Mechanistic or semimechanistic descriptions often benefit from nonlinear functions (Bolker, 2008) because their parameters have an ecologically meaningful interpretation (Miguez, Archontoulis, & Dokoohaki, 2017), helping to clarify system processes. Furthermore, ecological processes operate at multiple spatio‐temporal scales (Peters et al., 2008) producing data sets with hierarchical structures better handled by the use of random effects (Nakagawa & Schielzeth, 2013). Therefore, nonlinear mixed‐effects models can expand capabilities by including nonlinear regression and fixed and random effects (Lindstrom & Bates, 1990).

**Figure 1:**
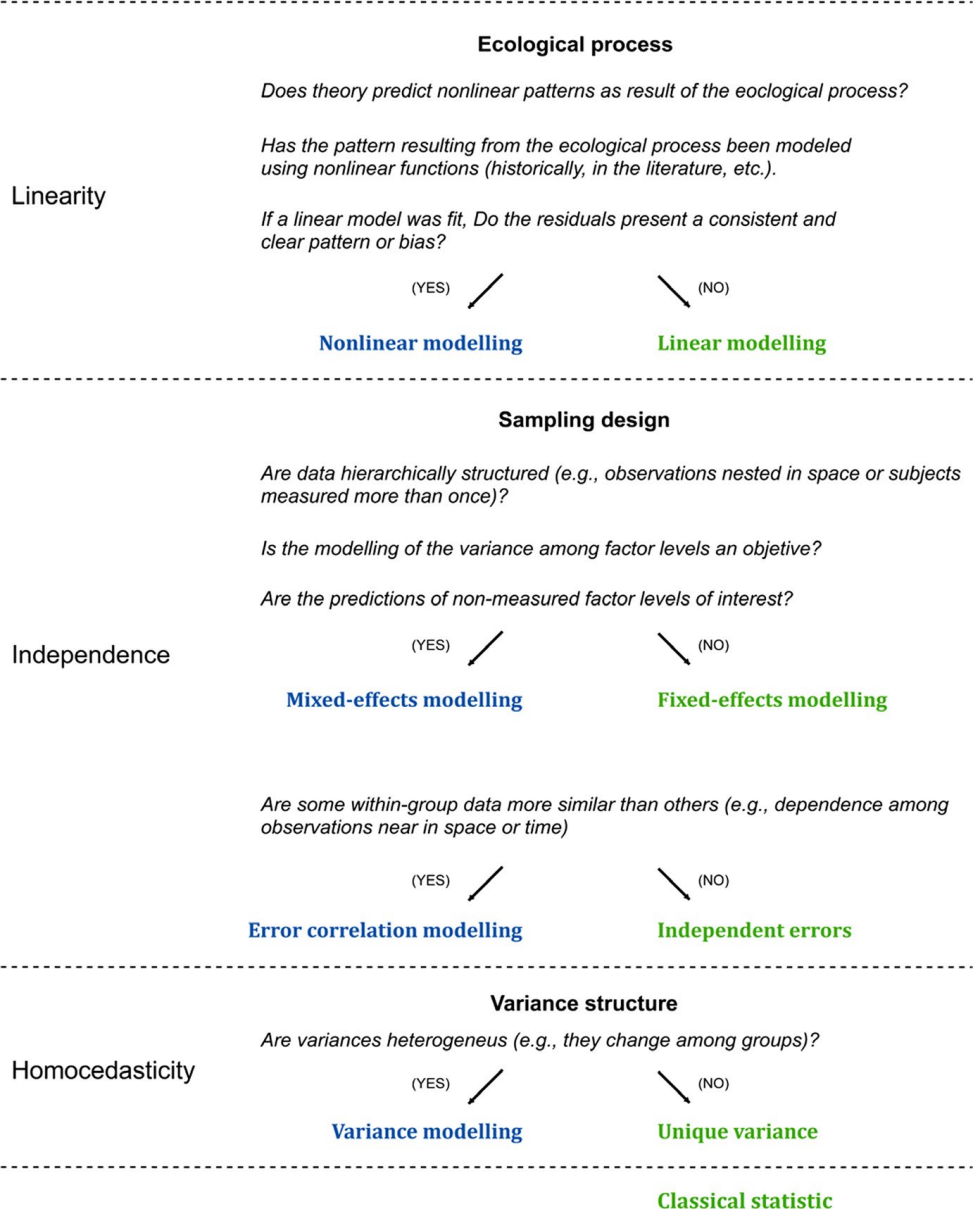
Decision‐tree scheme summarizing when the type of model introduced in this article would be useful to analyze ecological data. Decisions are categorized according to contexts linked to classical statistic assumptions (linear regression, ANOVA). Classical statistic is applied when linearity, independence, and homoscedasticity (and normality) can be guaranteed (green); otherwise, other approaches should be used (blue). This decision‐tree does not address all possible situations and approaches, for example, nonlinear patterns could be well modeled by using other linear approaches such as polynomials or generalized linear models [for more details, see Bolker (2008, p. 397)]

While nonlinear mixed‐effects models are not novel (Davidian & Giltinan, 2003), they still present several challenges to ecologists without formal training in statistics (Bolker et al., 2013). Some of these challenges arise from (a) the need to choose a suitable response function; there are many candidate functions and the variety can be overwhelming (Miguez et al., 2017); (b) patterns of correlation and variance usually occurs when experimental units (e.g., individuals, plots) are measured more than once (Davidian & Giltinan, 2003); (c) parameter estimation has no analytical solution and iterative methods must be applied (Bates & Watts, 2007) often leading to additional hurdles (e.g., provide reasonable starting values and model convergence; Bolker et al., 2013). While the last one represents a technical challenge, on the firsts two lies part of the answer to when or why to apply this complex modeling approach (Figure 1).

Regarding the first challenge, ecologists seek to match patterns to ecological theory or simply models to data (Richards, 2005). A large number of ecological process have nonlinear responses, and many deterministic functions have been proposed (a list of typical examples are found in Table 1). For instance, asymptotic patterns are commonly observed in ecology and could be described using rational functions (Bolker, 2008). One well‐known example comes from predator–prey dynamics (Kalinkat et al., 2013). Ecological theory predicts that per capita consumption rate of predators (*y*) varies with prey density (*x*) according to predator's capture rate and handling time (Rall et al., 2012). When capture rate (*a*) and handling time (*b*) are not supposed to vary with density, the pattern is called “type‐II functional response” (Holling, 1959) and mathematically formalized as *y* = *ax*/(1 + *abx*). In addition, it is possible to reparameterize response functions according to ecological questions (Bolker, 2008). For example, in the type‐II functional response as presented, the parameters to estimate are *a*, a measure of hunting efficiency or successful search, and *b*, which indicates the time used to kill, ingest, and digest a prey (Jeschke, Kopp, & Tollrian, 2002). However, we can rewrite *a* as 1/*a*′ and *b* as 1/*a*′*b*′ and re‐express the function as *y* = *a*′*x*/(*b*′ + *x*). Now, *a*′ represents the maximum per capita consumption rate reached when density is large (the asymptote of the function) and *b*′ is the density (*x*‐value) at the half‐maximum consumption rate. In fact, several strategies to address nonlinear patterns are widely used in ecology (e.g., transformations, polynomials, “splines”) but the main argument against those is that nonlinear models have meaningful parameters (other arguments are lack of parsimony and nonvalidity beyond range of fit; Pinheiro & Bates, 2000).

**Table 1:** Some common nonlinear patterns in ecology and (two possible) response functions for describing them [for a more complete list of nonlinear functions see Miguez et al. (2017)]

Pattern		Function		Ecological context
J‐shaped	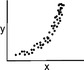	Exponential	*y* = *ae^bx^*	Population ecology (population growths without resource constrains). Eco‐physiology (temperature responses). Epidemiology (outbreaks).
		Power	*y* = *ax^b^*	
Saturating	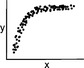	Michaelis–Menten	*y* = *ax/(b + x)*	Population ecology (type‐II functional response in predator–prey dynamics). Community ecology (resource competition). Eco‐physiology (photosynthetic curves). Forest ecology (light availability in canopy). Production ecology (fisheries, fruit quality). Epidemiology (infection rates).
		Monomolecular	*y* = *a*[1 − *e* ^−^ *^bx^*]	
S‐shaped (sigmoidal)	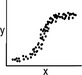	Logistic	$y=1/\left[1+e^{-\left(a+bx\right)}\right]$	Life history (individual biological growths). Population ecology (population growths with resource constrains, type‐III functional response in predator–prey dynamic). Forest ecology (stand dynamics).
		Gompertz	$y=ae^{-e^{-bx}}$	
Hump‐shaped (unimodal)	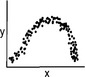	Ricker	*y = axe^bx^*	Population ecology (capture rates varying with prey size in predator–prey dynamic). Community ecology (richness species varying with productivity or disturbance gradients). Eco‐physiology (optimums). Fire ecology (fire activity along global productivity gradient).
		Beta	$y=a\left[1+\frac{b-x}{b-c}\right]{\left[\frac{x}{b}\right]}^{\frac{b}{b-c}}$	

Note

Terms in equations: *y* = response variable; *x* = explanatory variable; *e* = constant (the base of the natural logarithm); *a*, *b*, *c* = parameters. Parameter values must be in a certain range to match the pattern (e.g., power functions result J‐shaped curves when *b* > 1, but inverted J‐shaped if *b* < 0 and decreasing increments with 0 < *b *< 1). Function names and parameterization vary according to the context in which they are used (see Bolker, 2008).

Regarding the second challenge, when nonlinear ecological patterns are, as commonly occurs in ecology, observed from grouped data (e.g., observations spatially clustered, subjects measured more than once, individuals from the same family, species with phylogenetic relationships; Barnett, Koper, Dobson, Schmiegelow, & Manseau, 2010), mixed‐effects approaches allow correlations within‐group observations to be considered and modeling of heteroscedasticity (Davidian & Giltinan, 2003). For example, we might be interested in studying regional fruit production and designing an experiment where size of individual fruits was recorded over time (growth is usually sigmoidal), allowing for fruits to be nested in trees, trees in orchards, and orchards in regions. In such an experiment, a nonlinear mixed‐effects model would allow us to fit temporal curves to each fruit and to evaluate whether (some parameter of) the curves depend on fruit location, tree species, orchard management, or climate, all of these being incorporated in the model as predictors at different clustering levels (West, Welch, & Galeki, 2007). Indeed, one of the most intuitive applications of nonlinear mixed‐effects models is to describe temporal within‐individual responses and to identify factors determining variability among individual responses (Davidian & Giltinan, 2003). In short, nonlinear mixed‐effects models are nonlinear response functions allowing among‐groups random variation in (one or more) parameters which can be modeled from group‐level predictors (Bolker, 2008); these are convenient to apply with grouped data to describe a nonlinear ecological response, but their use entails more complexity than classical statistics.

Live vegetation moisture, termed as live fuel moisture content (LFMC) in the fire ecology context, is a typical ecological variable in which nonlinearity and correlation structures make it difficult to apply classical statistical methods. To burn, an ecosystem requires precipitation for plant biomass (fuel) to be produced, and dry weather conditions to make that biomass available for burning (Bradstock, 2010). LFMC is an ecological variable determining fuel biomass availability, thus influencing outbreak and spread of wildfires (Rossa, 2017). In other words, because removing the water from fuel requires energy (Jolly et al., 2012), higher moisture content means longer heating time to ignition and slower fire spread (Finney, Cohen, McAllister, & Jolly, 2013). Consequently, LFMC modeling becomes relevant in fire‐prone ecosystems, such as those of Mediterranean regions, where it is important to predict the vegetation moisture threshold at which fires are highly probable (Dennison & Moritz, 2009). Temporal changes in LFMC are determined by physiological and phenological factors associated with weather seasonality. This seasonality influences plant growth rates, water loss by transpiration, and changes in soil water availability (Nelson, 2001). Hence, in Mediterranean‐type climate regions (cold and humid winters, temperate and dry summers), such as northwestern Patagonia (Kottek, Grieser, Beck, Rudolf, & Rubel, 2006), seasonality causes plants to have a relatively high moisture during spring (when sprouting takes place) and then lower values during the autumn senescence (Keeley, Bond, Bradstock, Pausas, & Rundel, 2012). Therefore, LFMC is expected to reach a maximum during the growing season and steadily decrease through the dry season (when fires occur), until it stabilizes at a minimum; this is, naturally, a nonlinear response. In northwestern Patagonia, for example, vegetation growth season starts in early spring and ends in late summer/early autumn (Jobbágy, Sala, & Paruelo, 2002) overlapping part of the fire season, which starts in late spring/early summer (Oddi & Ghermandi, 2016). Since plants develop varied strategies to access water and regulate their water content status, LFMC modeling should consider how moisture seasonal variation differs among plant functional types (Castro, Tudela, & Sebastià, 2003). For example, in extra‐Andean Patagonia, grass and shrubs have different water‐use strategies (Sala, Golluscio, Lauenroth, & Soriano, 1989); shrubs obtain water from deeper soil layers (Golluscio & Oesterheld, 2007). Furthermore, phenological water‐use strategies within the same functional group can vary among coexisting species, as appears to occur with the shrubs *Mullinum spinosum* (Cav.) Pers. and *Senecio filaginoides* DC (Fernández, Nuñez, & Soriano, 1992), the first one with deeper root system (Fernandez & Paruelo, 1988), or along aridity gradients, in response to changes in water availability dynamics in soil (Golluscio & Oesterheld, 2007). Therefore, in this region, it is expected that parameters of the LFMC temporal curve depend on type of vegetation and aridity conditions. Lastly, because field sampling must consider LFMC within‐sampling location variability (Yebra et al., 2013), observations are commonly clustered in space (Desbois, Deshayes, & Beudoin, 1997). Hence, LFMC data obtained through field monitoring appear suitable to be analyzed using nonlinear mixed‐effects models.

Although nonlinear mixed‐effects approaches are useful in many areas (Davidian & Giltinan, 2003), including ecology and environmental sciences (Crecente‐Campo, Tomé, Soares, & Diéguez‐Aranda, 2010; Miguez, Villamil, Long, & Bollero, 2008), very few worked examples exists (Bolker et al., 2013). In order for statistical methods to be gradually applied by users, these must be demonstrated and illustrated with examples (Qian, Cuffney, Alameddine, McMahon, & Reckhow, 2010). Here, we illustrate the nonlinear mixed‐effects modeling approach using an ecological example involving the temporal dynamics of live vegetation moisture in northwest extra‐Andean Patagonia. From an ecological perspective, we apply a nonlinear mixed‐effects approach to model temporal changes in LFMC. In particular, we test if moisture content and drying pattern over the fire season differ (a) between grasses and shrubs, (b) between coexisting species, and (c) between sites with different aridity conditions. From a methodological point of view, we aimed to (a) describe a “step‐by‐step” statistical modeling process and (b) show that, compared to other linear and more classical approaches, nonlinear mixed‐effects models improve the description of ecological processes with seasonality such as temporal dynamics in LFMC. This “improvement” is assessed in terms of goodness of fit, model assumptions, and ecological meaning. We start with a nonlinear function linked to a simple statistical model, which is made increasingly complex to include the different sources of variability and correlation structures. While the modeling process is illustrated on LFMC, the framework is useful for other ecological variables with nonlinear patterns and correlation structures. To make this procedure easily reproducible, we provide the R codes used to perform the statistical analyses.

## 2 METHODS

### 2.1 Study area

**Figure 2:**
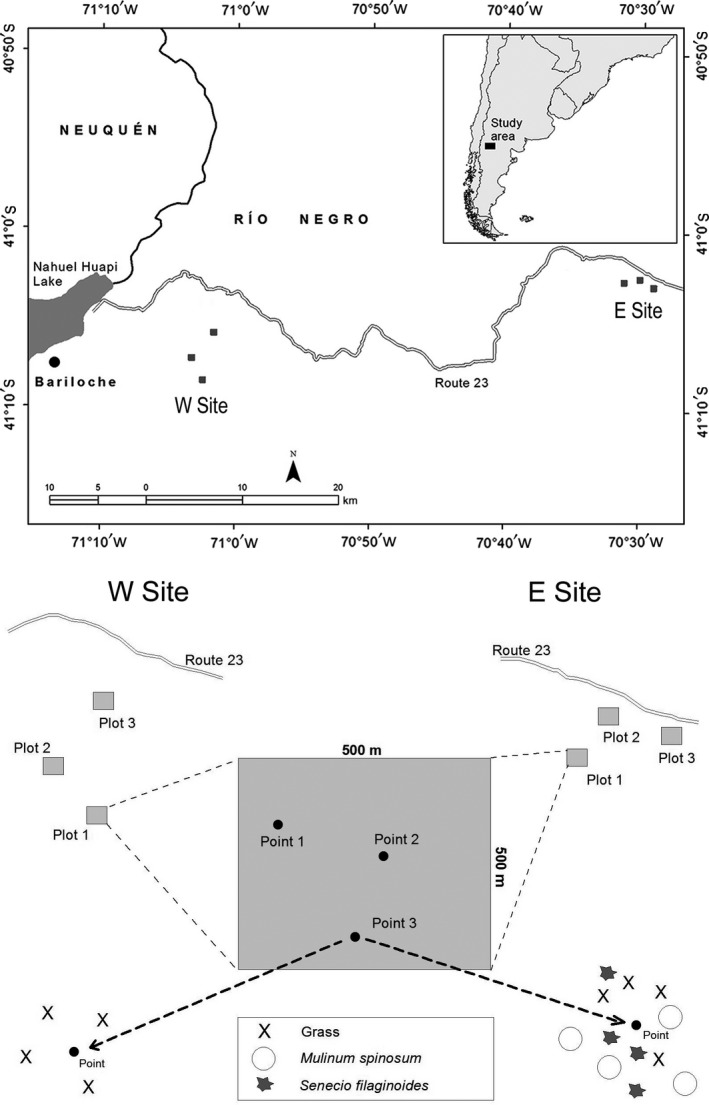
The upper map shows the study area (located in the northwestern region of extra‐Andean Patagonia, in Río Negro province, Argentina). At each sampling site, we established three plots (dark gray squares). The lower figure shows the detailed spatial sampling design. In each plot, we randomly selected three points and harvested biomass from the four nearest individuals to the point. Sites have different plant physiognomies: W site is an herbaceous steppe and E site is a grass–shrub steppe where *M. spinosum* and *S. filaginoides* are the main shrub species

Field data were gathered from northwestern Patagonia (east of Nahuel Huapi Lake, Río Negro, Argentina; Figure 2). The area is characterized by a semiarid climate with a Mediterranean‐type precipitation regime, and annual precipitation decreases in a steep 50‐km west–east gradient from 580 to 260 mm (San Ramón and INTA Pilcaniyeu weather stations). Along this climatic gradient, we established two sampling sites separated by 60 km (Figure 2) with different aridity and plant physiognomy. The western (W) site, the wettest, is a grass steppe dominated by the perennial grass *Festuca pallescens* (St. Yves) Parodi, and shrub cover is less than 5%. The eastern (E) site, the driest, is a shrub–grass steppe with 60% shrub cover where communities are codominated by *Papostipa speciosa* and by the shrubs *M. spinosum* and *S. filaginoides* (Godagnone & Bran, 2009).

### 2.2 Experimental design

We carried out the field sampling during 80 days (seven sampling dates distributed as evenly as possible) from 13 November 2013 to 10 February 2014. Due to the differences in the plant physiognomies of the sites, we measured LFMC in grasses at the W site and both grasses and shrubs (*M. spinosum* and *S. filaginoides*) in the E site. In both sites, we established three 500 × 500 m plots and average distance between plots was ≈2 km (Figure 1). All plots were near the road (National Road No. 23) (Figure 2) and on flat‐level terrain to avoid changes in LFMC caused by differences in topography. On each sampling date and plot, we randomly selected three spatial points (i.e., spatial locations within plots) for each leaf type (grasses and the two shrub species); we collected 80–100 g of live biomass from the nearest four individuals to these points (i.e., each observation came from a composite sample, Figure 2). Therefore, our total observations resulted in 252 measurements of LFMC (3 leaf types × 3 points × 3 plots × 7 sampling dates in the E site, plus 1 leaf type × 3 points × 7 sampling dates in the W site) from which five were lost (*n* = 247). This experimental design allowed us to compare plant functional types within the same site (grasses vs. shrubs in E site), sites within the same functional type (grasses in the W site vs. grasses in the E site), and species within the same functional type and site (*M. spinosum* vs. *S. filaginoides* in the E site).

We collected all samples between 12:00 and 16:00 hr local time. Immediately after collection, we packed the samples in individual hermetic plastic bags and transported them to the laboratory in a portable fridge. Once in the laboratory, we weighed the samples in a precision balance (0.01 g) to obtain their fresh weight (*W*_F_). Samples were oven‐dried at 80°C for 48 hr and reweighed to obtain their dry weight (*W*_D_). Finally, we calculated the LFMC (%) as: (*W*_F _− *W*_D_)/*W*_D_ × 100.

### 2.3 Nonlinear mixed‐effects model

**Figure 3:**
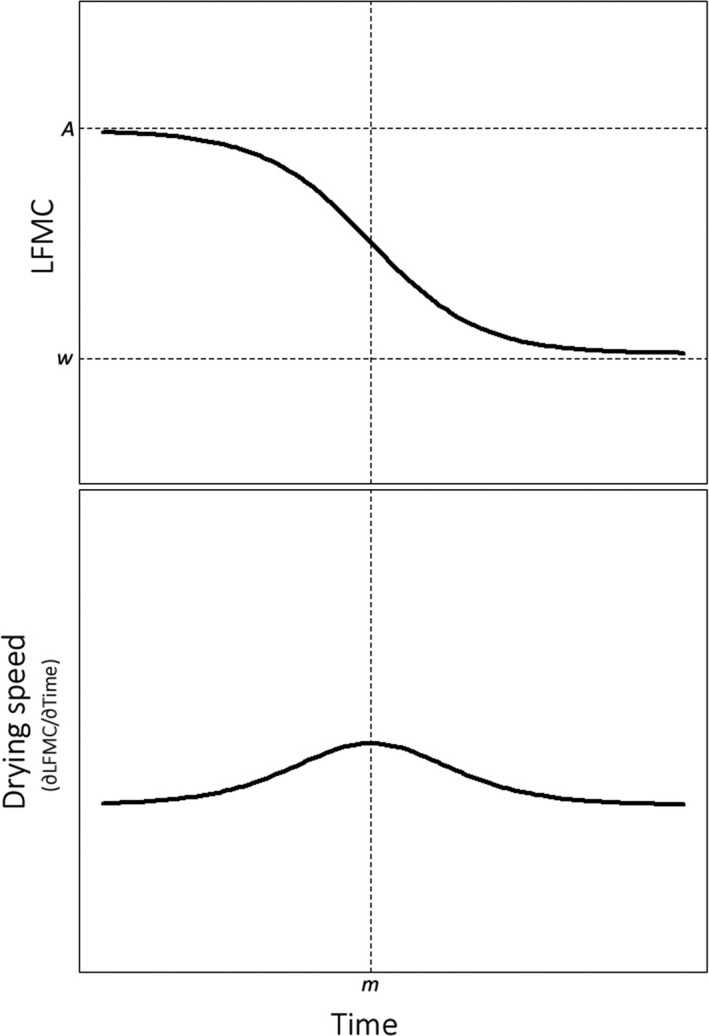
Live fuel moisture content (LFMC) as a function of time (*t*). According to a logistic‐type response function (top), LFMC is highest at the beginning of fire season (*A*) and decreases until it stabilizes at the end of season. The midway between *A* and *w* (point of inflection of the curve) occurs at *t* = *m*, when the drying speed, which is given by the first derivative of the logistic function (∂LFMC/∂*t*) (bottom), reaches a maximum

According to weather seasonality in northwestern Patagonia, LFMC should be maximum during spring and reach a minimum at the end of summer. Thus, as the fire season progresses, LFMC temporal patterns could be modeled with a declining logistic‐type function (Dennison et al., 2003), that is, a sigmoid and asymptotic curve. Since it was proposed by Verhulst (1938), different parameterizations have been used to model population growth and other physical or social features (Tsoularis, 2001). Among these, a flexible one is the four‐parameter logistic function (Pinheiro & Bates, 2000):

(1) $$y=\frac{A-w}{1+e^{\left(m-t\right)/s}}+w$$

where *y* and *t* are, respectively, the response and the predictor (time) variables, *e* is a constant (the base of the natural logarithm), *A* and *w* are respectively the upper and lower horizontal asymptotes, *m* is the value (time) at which *y* is midway between *A* and *w* (the inflection point), and *s* controls the curve steepness (Pinheiro & Bates, 2000). Applied to LFMC dynamics (Figure 3), *A* would represent the maximum LFMC reached during the growing season (when the peak occurs and shortly before the fire season starts), *w* the level at which the moisture is stabilized at the end of the fire season, *m* the time when the highest drying speed occurs (i.e., the maximum in the first derivative of the LFMC curve, Figure 3), and *s* is a parameter controlling the drying rate (it should be negative because LFMC is expected to decrease with time). Because LFMC is modeled as a function of time, the first derivative of the response function represents the instantaneous drying speed (∂LFMC/∂*t*):

(2) $$\frac{\partial \text{LFMC}}{\partial t}=\frac{\left(w-A\right)e^{\left(m+t\right)/s}}{s\left[2e^{\left(m+t\right)/s}+e^{2t/s}+e^{2m/s}\right]}$$

The resulting statistical model is:

(3) $${\text{LFMC}}_{i}∼N\left(\mu_{i};\sigma^{2}\right)$$

$$\mu_{i}=\frac{A-w}{1+e^{\left(m-t_{i}\right)/s}}+w$$

$$\text{Cor}({\text{LFMC}}_{i};{\text{LFMC}}_{i^{\prime }})=0$$

where *i* is the observation and, in our model, *t* is expressed as the number of days since the first measurement.

The parameters defining the nonlinear deterministic response function (*A*, *m*, *s*, *w*) in Equation 3 can vary among groups that could be considered as fixed or random effects (Pinheiro & Bates, 2000). Our aim is to understand if moisture content dynamic differs between grasses and shrubs, and between sites with different aridity conditions. Therefore, the next step was to include “leaf type” as a fixed effect with four levels: grasses in the W site (GW); grasses in the E site (GE); *M. spinosum* shrub in the E site (SM); *S. filaginoides* shrub in the E site (SS). The clustering imposed by the sampling design (observations grouped in plots) could lead to data with spatial correlation structure, that is, Cor(LFMC*_i_*, LFMC*_i′_*) ≠ 0 (Aarts, Verhage, Veenvliet, Dolan, & Sluis, 2014). Thus, plot was considered as a random effect to represent the correlation structure induced by the spatial nesting (Zuur et al., 2009). Hence, the new model takes into account that all parameters describing the temporal changes in LFMC vary with both leaf type (fixed effect) and plot (random effect). The nonlinear mixed‐effects model can be expressed as:

(4) $${\text{LFMC}}_{\mathrm{ji}}∼N\left(\mu_{i};\sigma^{2}\right)$$

$$\mu_{i}=\frac{A-w}{1+e^{\left(m-t_{i}\right)/s}}+w$$

$$A=A_{0j}+A_{1}{\text{GE}}_{i}+A_{2}{\text{SM}}_{i}+A_{3}{\text{SS}}_{i}$$

$$w=w_{0j}+w_{1}{\text{GE}}_{i}+w_{2}{\text{SM}}_{i}+w_{3}{\text{SS}}_{i}$$

$$m=m_{0j}+m_{1}{\text{GE}}_{i}+m_{2}{\text{SM}}_{i}+m_{3}{\text{SS}}_{i}$$

$$s=s_{0j}+s_{1}{\text{GE}}_{i}+s_{2}{\text{SM}}_{i}+s_{3}{\text{SS}}_{i}$$

$$\text{Cor}\left({\text{LFMC}}_{\mathrm{ji}};{\text{LFMC}}_{ji^{\prime }}\right)=\phi$$

$$A_{0j}∼N\left(\mu_{A_{0j}};\sigma_{A_{0}}^{2}\right);w_{0j}∼N\left(\mu_{w_{0}j};\sigma_{w_{0}}^{2}\right);m_{0j}∼N\left(\mu_{m_{0j}};\sigma_{m_{0}}^{2}\right);s_{0j}∼N\left(\mu_{s_{0j}};\sigma_{s_{0}}^{2}\right)$$

Of the multiples ways in which mixed‐effects models can be written, we have chosen that termed as “combining separate local regressions” (Gelman & Hill, 2007). We follow the Gelman and Hill's (2007) notation, who use subscript *i* to represent the smallest unit of observation, within‐plot observation (points) in our experimental design (*i* = 1, 2, …, 247); and *j* to indicate groups, plots in this case (*j* = 1, 2, …, 6). GE, SM, and SS are binary variables taking values 1 or 0, used to code leaf type (see Table 2 for interpretation of the associated terms) recorded for point *i*. With this notation, we try to establish a clear connection between mathematical expression and software output (Appendix S1). While this model considers observations (*i*) within plot *j* to be correlated (*ϕ* is termed as intraclass correlation, and estimated as a function of among‐groups and within‐groups variability; Aarts et al., 2014), residuals are assumed to be independent and normally distributed with homogeneous variances. Normal random effects (*A*_0_ *_j_*, *w*_0_ *_j_*, *m*_0_ *_j_*, *s*_0_ *_j_*) and independence between the within‐plot observations and random effects are also assumed.

**Table 2:** Akaike information criterion (AIC) for contrasting models of the temporal dynamics in live fuel moisture content (LFMC)

Model	Number of parameters	log‐Likelihood	AIC	∆AIC
Nonlinear mixed‐effects (M1)	15	−950.8	1,931.7	–
Linear mixed‐effects (M3)	15	−984.2	19,998.5	66.8
Nonlinear fixed‐effects (M2)	27	−1,015.6	2,085.2	153.6
Classical regression (M4)	25	−1,030.5	2,111.0	179.3
Null (M5)	2	−1,423.9	2,851.7	920.1

Note

AIC is a goodness of fit measure (likelihood or log‐likelihood) that penalizes for complexity (number of parameters).

However, because measurements near in time tend to be more similar than when far apart (Davidian & Giltinan, 2003), correlations usually arise in time series violating the independence assumption (Lindstrom & Bates, 1990). Such temporal dependence can be addressed from a mixed‐effects modeling framework (Zuur et al., 2009). We explored ARMA (autoregressive–moving average) structures for modeling temporal correlation (Pinheiro & Bates, 2000). ARMA temporal correlation structures have two components defining their order (*u*, *v*). In our model, the first component (*u*) indicates that the within‐plot observations at time *t* are modeled as a function of *s* previous times and are named “autoregressive” parameters (*ρ*). The second component (*v*) refers to the number of moving average parameters (*θ*) and states that these observations are modeled as a function of *v* previous noise (*η*). For example, an ARMA(1,1) model (i.e., *u* = 1 and *v* = 1) states that, in plot *j*, the moisture content at time *t* (LFMC*_ji_*_(_ *_t_*_)_) is influenced by that one at time *t*‐1 (LFMC*_ji_*_(_ *_t_*_−1)_) according to:

(5) $${\text{LFMC}}_{ji\left(t\right)}=f\left(\rho *{\text{LFMC}}_{ji\left(t-1\right)}+\theta *\eta_{t-1}+\eta_{t}\right)$$

In practice, these correlations are modeled on residuals (sometimes are called *R‐side effects*), in contrast to that induced by grouping (called *G‐effects*) which enter the model in terms of correlation of observations (Bolker, 2015). The statistical model is now expressed as:

(6) $${\text{LFMC}}_{\mathrm{ji}}∼N\left(\mu_{i};\sigma^{2}\right)$$

$$\mu_{i}=\frac{A-w}{1+e^{\left(m-t_{i}\right)/s}}+w$$

$$A=A_{0j}+A_{1}{\text{GE}}_{i}+A_{2}{\text{SM}}_{i}+A_{3}{\text{SS}}_{i}$$

$$w=w_{0j}+w_{1}{\text{GE}}_{i}+w_{2}{\text{SM}}_{i}+w_{3}{\text{SS}}_{i}$$

$$m=m_{0j}+m_{1}{\text{GE}}_{i}+m_{2}{\text{SM}}_{i}+m_{3}{\text{SS}}_{i}$$

$$s=s_{0j}+s_{1}{\text{GE}}_{i}+s_{2}{\text{SM}}_{i}+s_{3}{\text{SS}}_{i}$$

$$Cor\left({\text{LFMC}}_{ji\left(t\right)};{\text{LFMC}}_{ji^{\prime }\left(t\right)}\right)=\phi$$

$$\text{Cor}\left({\text{LFMC}}_{ji\left(t\right)};{\text{LFMC}}_{ji\left(t-1\right)}\right)=f\left({\text{ARMA}}_{\left[s,q\right]}\right)$$

$A_{0j}∼N\left(\mu_{A_{0j}};\sigma_{A_{0}}^{2}\right)$; $w_{0j}∼N\left(\mu_{w_{0j}};\sigma_{w_{0}}^{2}\right)$; $m_{0j}∼N\left(\mu_{m_{0j}};\sigma_{m_{0}}^{2}\right)$; $s_{0j}∼N\left(\mu_{s_{0j}};\sigma_{s_{0}}^{2}\right)$

It is also important to consider the variance pattern (Davidian & Giltinan, 2003); ecological variables are often heteroscedastic (Bolker et al., 2013), and, many times, variance components are biologically as important as mean values (Schielzeth & Nakagawa, 2013). We used variance functions (components of a model with Gaussian distribution that allows for heterogeneity) to model LFMC variability at the within‐plot level ($\sigma_{i}^{2}$) as a function of leaf type:

(7) $${\text{LFMC}}_{\mathrm{ji}}∼N\left(\mu_{i};\sigma_{i}^{2}\right)$$

$$\mu_{i}=\frac{A-w}{1+e^{\left(m-t_{i}\right)/s}}+w$$

$$\sigma_{i}^{2}=f\left(\text{leaf}\;\text{type}\right)$$

$$A=A_{0j}+A_{1}{\text{GE}}_{i}+A_{2}{\text{SM}}_{i}+A_{3}{\text{SS}}_{i}$$

$$w=w_{0j}+w_{1}{\text{GE}}_{i}+w_{2}{\text{SM}}_{i}+w_{3}{\text{SS}}_{i}$$

$$m=m_{0j}+m_{1}{\text{GE}}_{i}+m_{2}{\text{SM}}_{i}+m_{3}{\text{SS}}_{i}$$

$$s=s_{0j}+s_{1}{\text{GE}}_{i}+s_{2}{\text{SM}}_{i}+s_{3}{\text{SS}}_{i}$$

$$\text{Cor}\left({\text{LFMC}}_{ji\left(t\right)};{\text{LFMC}}_{ji^{\prime }\left(t\right)}\right)=\phi$$

$$\text{Cor}\left({\text{LFMC}}_{ji\left(t\right)};{\text{LFMC}}_{ji\left(t-1\right)}\right)=f\left({\text{ARMA}}_{\left[s,q\right]}\right)$$

$A_{0j}∼N\left(\mu_{A_{0j}};\sigma_{A_{0}}^{2}\right)$; $w_{0j}∼N\left(\mu_{w_{0j}};\sigma_{w_{0}}^{2}\right)$; $m_{0j}∼N\left(\mu_{m_{0j}};\sigma_{m_{0}}^{2}\right)$; $s_{0j}∼N\left(\mu_{s_{0j}};\sigma_{s_{0}}^{2}\right)$

Specifically, we used *varIdent* as variance function (Pinheiro & Bates, 2000). In a *varIdent* function, the groups of a stratification variable (e.g., leaf types) are allowed to have different variance:

(8) $$\sigma_{i}^{2}={\left(\sigma_{\text{base}}{\text{GW}}_{i}+\sigma_{\text{base}}\delta_{1}{\text{GE}}_{i}+\sigma_{\text{base}}\delta_{2}{\text{SM}}_{i}+\sigma_{\text{base}}\delta_{3}{\text{SS}}_{i}\right)}^{2}$$

where *σ*_base_ is the standard deviation in the W site, and *δ*_1_, *δ*_2_, *δ*_3_ are the quotients between the standard deviation of the respective leaf types and *σ*_base_.

The proposed nonlinear mixed‐effects model, therefore, relaxes three of the four assumptions of classical regression (Figure 1): linearity (through the logistic‐type response function), homogeneity (through the variance function), and independence (through the random effects—spatial clustering—and the ARMA model—temporal correlation structure):

(9) $${\text{LFMC}}_{\mathrm{ji}}∼N\left(\mu_{i};\sigma_{i}^{2}\right)$$

$$\mu_{i}=\frac{A-w}{1+e^{\left(m-t_{i}\right)/s}}+w$$

$$\sigma_{i}^{2}={\left(\sigma_{\text{base}}{\text{GW}}_{i}+\sigma_{\text{base}}\delta_{1}{\text{GE}}_{i}+\sigma_{\text{base}}\delta_{2}{\text{SM}}_{i}+\sigma_{\text{base}}\delta_{3}{\text{SS}}_{i}\right)}^{2}$$

$$A=A_{0j}+A_{1}{\text{GE}}_{i}+A_{2}{\text{SM}}_{i}+A_{3}{\text{SS}}_{i}$$

$$w=w_{0j}+w_{1}{\text{GE}}_{i}+w_{2}{\text{SM}}_{i}+w_{3}{\text{SS}}_{i}$$

$$m=m_{0j}+m_{1}{\text{GE}}_{i}+m_{2}{\text{SM}}_{i}+m_{3}{\text{SS}}_{i}$$

$$s=s_{0j}+s_{1}{\text{GE}}_{i}+s_{2}{\text{SM}}_{i}+s_{3}{\text{SS}}_{i}$$

$$\text{Cor}\left({\text{LFMC}}_{ji\left(t\right)};{\text{LFMC}}_{ji^{\prime }\left(t\right)}\right)=\phi$$

$$\text{Cor}\left({\text{LFMC}}_{ji\left(t\right)};{\text{LFMC}}_{ji\left(t-1\right)}\right)=f\left({\text{ARMA}}_{\left[s,q\right]}\right)$$

$A_{0j}∼N\left(\mu_{A_{0j}};\sigma_{A_{0}}^{2}\right)$; $w_{0j}∼N\left(\mu_{w_{0j}};\sigma_{w_{0}}^{2}\right)$; $m_{0j}∼N\left(\mu_{m_{0j}};\sigma_{m_{0}}^{2}\right)$; $s_{0j}∼N\left(\mu_{s_{0j}};\sigma_{s_{0}}^{2}\right)$

Equation 9 refers to the more complex or global model, which includes variance modeling, temporal correlation, and fixed‐effects (leaf type) and random effects (plot) on all parameters of the response function. Nevertheless, not all of these components necessarily need to be in the model. If any of them is not important but included (the predictive capacity is not increased), the model will be overparameterized (Aho, Dewayne, & Peterson, 2014), which could cause convergence problems (Grueber, Nakagawa, Laws, & Jamieson, 2011). When convergence problems occur, reducing the model complexity is a possible solution (Bolker et al., 2009).

Therefore, we followed the Zuur's et al. (2009) protocol for fitting mixed‐effects models, first assessing the random structure and then the fixed effects. We compared models of different complexity level by using the Akaike information criterion (AIC), a goodness‐of‐fit measure (likelihood) that penalizes for complexity (number of parameters; see next section for more detailed discussion about AIC and multimodel inference). Although the model with the lowest AIC value can be chosen as the best one (Burnham & Anderson, 2002), in order to consider model uncertainty, differences in AIC should be large enough (Richards, 2005). We used delta AIC > 2, but other cutoffs could be chosen as rule of thumb (Harrison et al., 2018). To fit the model (see the R code), we first explored the variance–covariance structure (Barnett et al., 2010; West et al., 2007). Then, we evaluated if any of the four parameters (*A*, *w*, *m*, *s*) varied across the plots through random effects. For instance, to determine whether the maximum LFMC varied with plot we compared the model where *A* is random (*A*_0_ *_j_* in Equation 9) with the model where *A* is unique for all the plots (*A*_0_). Later, we decided about the inclusion of the temporal correlation and variance functions assessing whether the data fit obtained from the model introduced in Equation 4 were improved by that from Equations 6 and 9. Lastly, we modeled the fixed effects examining what parameters of the response function varied with leaf type. For instance, to assess if leaf type influences the maximum LFMC we compared the model in which *A* depends on leaf type (*A* = *A*_0_ + *A*_1_GE + *A*_2_SM + *A*_3_SS) with the model where *A* is unique (i.e., general to all leaf types). When the effect of leaf type on any parameter was found important (i.e., delta AIC > 2), we assessed differences among its levels (our ecological question).

### 2.4 Alternative models

We fitted four alternative models, which were compared to the nonlinear mixed‐effects model. The first one was a (logistic‐type) nonlinear fixed‐effects model. In this case, time, leaf type, and plot are treated as fixed effects, and normality and independence among data are assumed (M2). The second alternative (M3) was a linear mixed‐effects model with time and leaf type (and its interaction) as fixed effects and plot as random effect (spatial nesting). This model included an ARMA temporal structure and the same variance function as the one used in the nonlinear mixed‐effects model. The third alternative model (M4) was a classical regression (i.e., assuming that the relation with time is linear, residuals follow a normal distribution and data are independent in space and time and show homogeneous variances). We also fitted a null model (i.e., without predictors, M5) which was considered as the baseline in the comparisons. In short, we compared five models: nonlinear mixed‐effects (M1), nonlinear fixed‐effects (M2), linear mixed‐effects (M3), classical regression (M4), and null (M5). M1 and M2 share the deterministic response function (logistic‐type) but differ in the stochastic structure; similarly, M3 and M4 share a linear deterministic response function but differ in the stochastic structure. M1 and M3 share the random structure (mixed‐effects) and differ in the type of temporal relationship assumed (nonlinear vs. linear), similarly, M2 and M4 share the random structure and differ in temporal relationship assumptions. Before comparison, each alternative model was fitted according to parsimony, just as the nonlinear mixed‐effects model (M1).

Model comparison was carried out under a multimodel inference framework (Burnham & Anderson, 2002) using the AIC (Burnham, Anderson, & Huyvaert, 2011). This inference framework is especially suitable for selecting among non‐nested models (Burnham & Anderson, 2004); the fitting process of each model also involved model comparison but in this case, they were nested (see previous section). Our modeling approach was carried out using AIC as an index of parsimony but other related statistics exist (modifications of AIC, such as AICc, and others, such as BIC or DIC). All of these criterions share a similar goal, that is, to find a balance between goodness of fit and complexity. They have advantages and limitations and should be chosen according to the context and needs (statistical paradigm, assumptions, data structure; Barnett et al., 2010). For instance, Bayesian approaches could provide advantages when fitting complex models with few data points by incorporating prior distribution for parameters (Davidian & Giltinan, 1995; Gelman et al., 2013). Our approach, however, is useful beyond the information criteria chosen to select models (for further discussions, see Aho et al., 2014; Murtaugh, 2009; Richards, 2005; Spiegelhalter, Best, Carlin, & Linde, 2014; Yang, 2005).

The models were fitted using the nlme(), lme(), and gls() functions of the *nlme* package (Pinheiro, Bates, DebRoy, Sarkar, & Core Team, 2016) in R 3.3.5 (R Core Team, 2017). When the effects of leaf type were found important, we used the emmeans() function of the *emmeans* package (Lenth, 2018). Pearson residuals were visually assessed (residuals vs. fitted values plot, residuals vs. predictors plot, and normal Q‐Q plot) for checking the models' assumptions. The analysis is available in the Supplementary Material.

## 3 RESULTS AND DISCUSSION

### 3.1 Model comparison

**Figure 4:**
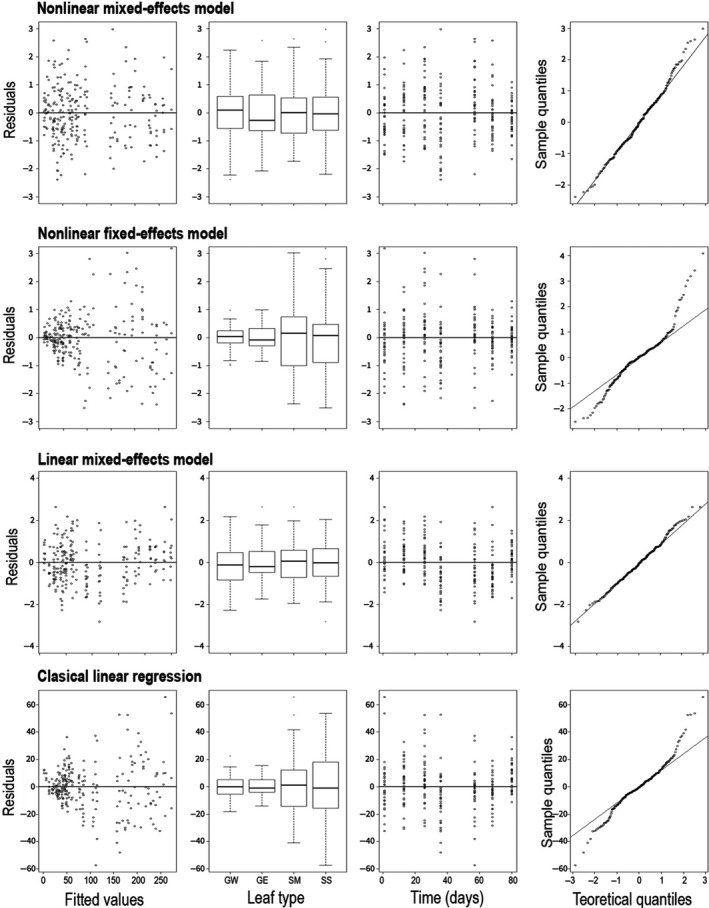
Residual analyses to evaluate model assumptions. In both mixed‐effects models (nonlinear and linear), residuals indicate that the model assumptions are reasonable. On the contrary, in the fixed‐effects models, assumptions are violated. Mixed‐effects models, which include correlation structures and variance modeling, remove the variance heterogeneity (standardized residuals vs. fitted values and vs. leaf type), the temporal autocorrelation (standardized residuals vs. time), and the lack of normality (observed vs. normal quantiles). The four levels of the leaf‐type factor are grasses in the site W (GW); grasses in the site E (GE); M. spinosum shrub (SM); S. filaginoides shrub (SS)

We found stronger support (lower AIC) for the nonlinear mixed‐effects model with these datasets (M1, Table 2). Both the residual analysis and the visual analysis of the fitted curves confirm a temporal pattern of LFMC during the fire season to be well‐described by a declining logistic‐type function. Indeed, the (nonlinear) deterministic component of this model was enough to capture all the temporal changes suggesting it is not necessary to include the temporal correlation structure. According to the residual pattern (Figure 4), assumptions are reasonable under the linear mixed‐effects model (M3). In this model, however, the LFMC nonlinear temporal pattern was captured by the residuals' autocorrelation (ARMA). On the other hand, neither the nonlinear fixed‐effects model (M2) nor the classical regression (M4) was appropriate to model temporal changes in LFMC (the residual analyses show violation to the assumptions of these models, see Figure 4).

The fact that both linear and nonlinear mixed‐effects models were suitable highlights the importance for variables such as LFMC to be addressed from a mixed‐effects modeling approach. Nonetheless, the nonlinear strategy was better than the linear one. While the general mixed‐effects approach accounts for spatial nesting, temporal dependence, and heterogeneity among leaf types, the nonlinear response function adds the capability to model seasonal patterns. In this regard, the logistic‐type model answers questions such as “what is the minimum moisture content of a species and when is it reached?” and enables estimation of the instantaneous drying speed (in a linear model it is only possible to know the average drying speed; the first derivative is constant; Paine et al., 2012). Hence, the nonlinear temporal relationship is not only underpinned by data but is also conceptually more relevant. In fact, nonlinear approaches allow statistical models based on physical, biological or ecological ideas (Jonsson et al., 2014).

### 3.2 Interpreting the nonlinear mixed‐effects model

**Figure 5:**
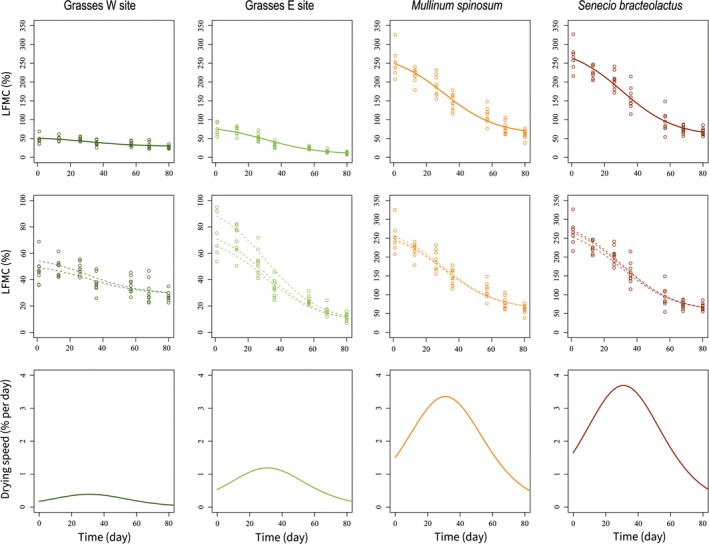
Overall (upper panels) and plot‐level (medium panels) predictions from the nonlinear mixed‐effects model (M1) for each leaf type. The drying rate (lower panels) was obtained analytically as the first derivative of each logistic‐type curve. The x‐axis shows the number of days since the first measurement (13 November 2013), which was close to the beginning of the fire season

Nonlinear functions provide an interesting approach to understanding the temporal dynamics of ecological variables (Pascual & Ellner, 2000). The proposed logistic‐type curve described LFMC temporal changes (Figures 4 and 5) using four parameters (*A, w*, *m*, *s*) varying with leaf type as a fixed effect. LFMC of both shrubs and grasses decreased from mid‐spring to summer, tracking the temporal trend of the Mediterranean‐type precipitation regime. The same overall sigmoidal pattern is observed for all of the leaf types, although considerable variation among them exists (Figure 5). In particular, our modeling effort suggests that leaf types differed in their maximum (*A*) and minimum LFMC (*w*) (Table 3). In Patagonian steppes, soil moisture increases with depth (Sala et al., 1989) and water from deeper soil layers is available for longer periods than shallow water since it is less affected by evaporative demand (Ferrante, Oliva, & Fernández, 2014). Because shrubs can reach water from deeper soil layers (Golluscio & Oesterheld, 2007), higher LFMC in shrubs than grasses should be expected. Accordingly, shrubs moisture content at the beginning of the fire season ($\hat{A}$) was, on average, four times higher than for grasses and the stabilization value ($\hat{w}$) was three times higher (Figure 5, Table S4). This implies different drying speed (slopes) between the functional groups, as inferred from the derivatives of their temporal curves (Figure 5).

**Table 3:** Parameter estimates with their units (95% confidence intervals in square brackets) and their meaning for the fitted nonlinear mixed‐effects model of live fuel moisture content (LFMC)

Parameter	Estimate	Meaning
$\mu_{A_{0}}$	54.3% [42.5:66.0]	Maximum LFMC ($\hat{A}$) of grasses in the W site. A varies with plot and therefore a hyperparameter is estimated.
*A*_1_	30.9% [13.5:48.3]	Difference between $\hat{A}$ of grasses in the E site and $\hat{A}$ of grasses in W site.
*A*_2_	223.4% [191.9:254.6]	Difference between $\hat{A}$ of *M. spinosum* and $\hat{A}$ of grasses in the W site.
*A*_3_	240.3% [207.1:273.5]	Difference between $\hat{A}$ of *S. filaginoides* and $\hat{A}$ of grasses in the W site.
*w*_0_	29.1% [25.7:32.4]	Minimum LFMC ($\hat{w}$) of grasses in the W site.
*w*_1_	−20.7% [−25.8: −15.6]	Difference between $\hat{w}$ of grasses in the E site and $\hat{w}$ of grasses in the W site.
*w*_2_	31.7% [16.4:47.0]	Difference between $\hat{w}$ of *M. spinosum* and $\hat{w}$ of grasses in the W site.
*w*_3_	26.7% [11.1:42.9]	Difference between $\hat{w}$ of *S. filaginoides* and $\hat{w}$ of grasses in the W site.
*m*	30.9 days [26.9:35.0]	Day when the LFMC half‐maximum occurs ($\hat{m}$) in the study area (data suggest one general value for all the leaf types).
*s*	−16.1 [−20.8: −11.5]	Parameter controlling speed of change ($\hat{S}$) of the LFMC (data suggest one general value for all the leaf types, the negative value indicates vegetation to be drying during the fire season).
$\sigma_{\text{base}}$	7.1% [6.0:8.6]	LFMC variability within‐plots (residual standard error) of grass (${\hat{\sigma }}_{\text{gw}}$) in Site W at the beginning the fire season.
*δ*_1_	0.9 [0.7:1.2]	Ratio between the within‐plots standard error of the LFMC of grasses in Site E (${\hat{\sigma }}_{\text{ge}}$) and grasses in W site (${\hat{\sigma }}_{\text{gw}}$)
*δ*_2_	3.2 [2.4:4.0]	Ratio between the within‐plots standard error of the initial LFMC *M. spinosum* (${\hat{\sigma }}_{\text{sm}}$) and grasses in W site (${\hat{\sigma }}_{\text{gw}}$)
*δ*_3_	3.0 [2.4:3.9]	Ratio between the within‐plots standard error of the initial LFMC *S. filaginoides* (${\hat{\sigma }}_{\text{ss}}$) and grasses in W site (${\hat{\sigma }}_{\text{gw}}$)
$\sigma_{A_{0}}$	9.4% [3.9:15.8]	Variability among plots (standard deviation) in the maximum LFMC (random effects on *A*).

Grass LFMC dynamics differed between sites (Figure 5). Specifically, grasses showed different saturation moisture and minimum moisture content (*A*_1_ ≠ 0, and *w*_1_ ≠ 0, respectively; see Table 3 and Appendix S1 to interpret both parameters). These different responses could be caused by environmental differences between sites and/or functional differences between grass species. At the beginning of the study (mid‐spring, when water availability begins to decrease in Patagonia; Sala et al., 1989), moisture content was higher in grasses from the E site ($\hat{A}$ = 85%) than that from the W site ($\hat{A}$ = 54%). The 95% confidence interval for such difference (31%) in the maximum LFMC between the grasses of the both sites (*A*_1_) spanned from 13% to 48% (Table 3), reflecting the degree of uncertainty in the true value of the point estimate. While the W site is dominated by *F. pallescens*, the dominant grass in the E site is *P. speciosa*. Both species have xerophytic foliar traits associated to resistance to water stress (Latour, 1979), but *P. speciosa* has more convoluted blades and stomatal crypts with higher trichome density (L. Ghermandi, data not published) and thus would prevent water loss more efficiently. In addition, in arid and semiarid areas, shrubs act as thermal buffers, increasing water availability (Villagra et al., 2011). Hence, shrub presence could benefit superficial soil water availability in the E site (shrubs are not present in the W site), increasing grass LFMC. These factors could allow grasses at the E site to maintain relatively high moisture during the initial phase of water stress (i.e., higher *A*; Figure 5). However, as the dry period (i.e., the fire season) progresses, greater aridity at the E site could overcome the initial advantages. The model suggests higher drying speed in site E grasses, mainly in the middle of the fire season (Figure 5), causing late December LFMC to become lower than that of grass growing in the W site (i.e., lower *w*; Figure 5). The effect of shrubs on the grasses LFMC dynamics, and thus on fire probability, could be other of the ecological interactions commonly observed in Patagonia between these two functional groups (Cipriotti, Aguiar, Wiegand, & Paruelo, 2014; Gonzalez & Ghermandi, 2019). Although both shrubs species have different root systems (Fernandez & Paruelo, 1988), their LFMC dynamics appear similar ($\hat{A}$ = 295%, $\hat{w}$ = 56% in *S. filaginoides*, and $\hat{A}$ = 278%, $\hat{w}$ = 61% in *M. spinosum*) (Figure 5). Again, it is worthy to recognize uncertainty around estimates (*A*_2_ and *A*_3_ in this case) and, hence, given the confidence intervals (Table 3).

In contrast to that observed in *A* and *w*, the steepness of the curves (*s*) was similar for all the leaf types and the time when the drying was highest (*m*) occurred simultaneously (31 days since beginning of the experiment). In other words, it is reasonable to assume *s*_1_, *s*_2_, *s*_3_, *m*_1_, *m*_2_, and *m*_3_ (Equation 9) to be zero. These results suggest absolute levels of moisture content to be leaf‐type‐specific while the drying rate could be a functional trait operating at ecosystem or plant community level. In ecological terms, selecting a simpler model implies that we are treating the different leaf types as behaving as a group with similar drying responses.

Mixed‐effects models allow us to understand and predict ecological variables at different hierarchies (Qian et al., 2010). In our example, the proposed model considered LFMC temporal curves varying with plot as a random effect (Figure 5); the results indicate that the random effect of plot was only important for *A* (i.e., $\sigma_{w_{0}}^{2}=\sigma_{m_{0}}^{2}=\sigma_{s_{0}}^{2}=0$). The later, along with the fact that plots had similar minimum LFMC (i.e., data suggested *w* not to vary with plot), could have implications for the behavior of fires occurring at different times along the fire season. Fires occurring at the beginning of the season (higher LFMC variability) should be more heterogeneous and less severe than at the end, when LFMC is lower and its spatial pattern is less variable. Nonetheless, because vegetation water status responds to rainfall variability, particularly in arid and semiarid regions such as extra‐Andean Patagonia (Golluscio, Sigal Escalada, & Pérez, 2009), LFMC dynamics models should incorporate interannual variability in precipitation as an explanatory variable. While our sampling period covered only one fire season, the proposed model allows adding precipitation (or other climatic variable) as a fixed effect (*A*, *w*, *m*,* s* = *f*[precedent precipitation]) or via a random effect (incorporating year (*k*) as an additional hierarchy: *A_0k_*, *w_0k_*, *m_0k_*, *s_0k_*) (Bolker, 2015). Both strategies would represent different conceptual models. If precipitation is incorporated as a fixed effect (i.e., a covariate), the model would be rather mechanistic (we would model the effect of water availability). If precipitation gets into the model as a random effect of year, we would estimate among‐years variability due to climatic differences. In addition, it would be possible to incorporate a plot‐level predictor (e.g., productivity) to model spatial variability in parameters at this level ($\mu_{A_{0j}},\mu_{w_{0j}},\mu_{m_{0j}},\mu_{s_{0j}}$ = *f*[plot productivity]) or even to add other spatial hierarchies (e.g., site) and to quantify spatial variability at greater scales. The unexplained variance could be reduced because of adding a plot‐level predictor, allowing more precise estimation of fixed effects (Schielzeth & Nakagawa, 2013).

Variance heterogeneity is expected in many ecological variables (Benedetti‐Cecchi, 2003). Within‐plot LFMC variability was three times higher in shrubs than in grasses but was similar between grasses from the two sites (${\hat{\delta }}_{1}$ = 0.97) and between the two shrub species (${\hat{\delta }}_{2}$ = 2.79; ${\hat{\delta }}_{3}$ = 3.17). Certainly, the model could be further simplified by applying a two‐level variance function (grasses/shrubs, that is, $\sigma_{i}^{2}$ as a function of plant functional type or growth form). The observed heteroscedasticity between growth forms could respond to differences in LFMC (higher values in shrubs), as commonly the variances tend to increase with the mean of the response variable. However, it could be also related to environmental conditions such as more homogeneous soil water availability for grasses than for shrubs (Golluscio & Oesterheld, 2007). In fact, variation in water availability is minimal in superficial soil layers (where grasses obtain the water) and increases with depth (Bucci, Scholz, Goldstein, Meinzer, & Arce, 2009). In addition, variance components of a LFMC mixed‐effects model could contain relevant information for planning field sampling linked to vegetation moisture monitoring from remote sensing. For instance, plot‐level information should be prioritized if random‐effect variance was significantly larger than residual variance (Schielzeth & Nakagawa, 2013). Here, random‐effect variance (among‐plot variability) was quantified from the standard deviation of *A* (${\hat{\sigma }}_{A_{0}}$ = 9%), which was similar to intraplot standard deviation of grasses from the W site (${\hat{\sigma }}_{\text{base}}$ = 7%). This type of information is critical for developing sampling protocols that improve estimates of plant moisture content (Yebra et al., 2013) and other seasonal variables (Watson, Restrepo‐Coupe, & Huete, 2019) from satellite images.

## 4 CONCLUSIONS

Our work covers a poorly addressed topic in ecology: illustrate the statistical modeling process using a nonlinear mixed‐effects framework. Similar to many other ecological variables, time series of vegetation moisture do not fit into classical statistical methods. We applied a nonlinear approach to model vegetation moisture dynamics proposing a logistic‐type function based on ideas about the dynamics of the system. Our model had greater support than alternative (and less complex) models. Parameter interpretation can be linked to vegetation features and environmental conditions showing how nonlinear mixed‐effects models could be used to advance ecological theory and practice. For instance, we addressed ecological questions about LFMC dynamic of grasses and shrubs under different aridity conditions, which could have applications in fire management. In this respect, we encourage researchers to propose statistical models based on conceptual ideas rather than adjusting data to standard models that many times involve data transformation to meet model assumptions. Due to the lack of worked examples in the literature, our approach can be useful to researchers addressing different ecological problems.

## CONFLICT OF INTEREST

None declared.

## AUTHORS' CONTRIBUTIONS

FJO and LG conceived the ideas and designed the sampling design; FJO collected the data; FJO, FEM, and LOB analyzed the data; FEM developed the selfStart function; FJO and FEM wrote the R scripts; FJO and LAG led writing of the manuscript; and all authors contributed critically to drafts.

## Data Availability

We provided R codes and data (“data_lfmc.txt”) in Supporting Information. The codes include a self‐starting function (“s1.R”) along with the fit of the nonlinear mixed‐effects model, the fit of the alternative models, and model comparison (“s2.R”).
