# Supplementary material for: A nonlinear mixed‐effects modeling approach for ecological data: Using temporal dynamics of vegetation moisture as an example
Source: Ecol Evol. 2019 Aug 15;9(18):10225–40. doi: 10.1002/ece3.5543 (PMC6787861; doi:10.1002/ece3.5543)
Supplement: Supplementary file 1 [file ECE3-9-10225-s001.pdf]

Appendix A1

Here we show how to extract the parameter estimates of the nonlinear mixed-effects model from the R output. For this, the statistical model (1), R output (2), and table of results presented in the manuscript (3), are linked in a new table (4).

(1) Nonlinear mixed-effects model (equation 9 in manuscript, page 12):

$$\mathrm{LFMC}_{ji} \sim \mathcal{N}(\mu_i; \sigma^2)$$

$$\mu_i = \frac{A - w}{1+e^{(m - t_i)/s}} + w$$

$$\sigma_i^2 = (\sigma_{base}\mathrm{GW}_i + \sigma_{base}\delta_1\mathrm{GE}_i + \sigma_{base}\delta_2\mathrm{SM}_i + \sigma_{base}\delta_3\mathrm{SM}_i)^2$$

$$A = A_{0j} + A_1\mathrm{GE}_i + A_2\mathrm{SM}_i + A_3\mathrm{SS}_i$$

$$w = w_{0j} + w_1\mathrm{GE}_i + w_2\mathrm{SM}_i + w_3\mathrm{SS}_i$$

$$m = m_{0j} + m_1\mathrm{GE}_i + m_2\mathrm{SM}_i + m_3\mathrm{SS}_i$$

$$s = s_{0j} + s_1\mathrm{GE}_i + s_2\mathrm{SM}_i + s_3\mathrm{SS}_i$$

$$Cor(\mathrm{LFMC}_{ji}; \mathrm{LFMC}_{ji'}) = f(ARMA_{[s,q]})$$

$$A_{0j} \sim \mathcal{N}(\mu_{A_{0j}}; \sigma_{A_0}^2) \;\; ; \;\; w_{0j} \sim \mathcal{N}(\mu_{w_{0j}}; \sigma_{w_0}^2) \;\; ; \;\; m_{0j} \sim \mathcal{N}(\mu_{m_{0j}}; \sigma_{m_0}^2) \;\; ; \;\; s_{0j} \sim \mathcal{N}(\mu_{s_{0j}}; \sigma_{s_0}^2)$$

(2) R output (line 163 in script s2.R)

> M1 <- update(nl.me.ml.2, method = "REML")

> summary(M1)

```
Nonlinear mixed-effects model fit by REML
  Model: lfmc ~ SSdlf(time, A, w, m, s)
 Data: lfmc.data.gd
       AIC      BIC   logLik
  1931.668 1983.689 -950.834

Random effects:
 Formula: A ~ 1 | group
        A.(Intercept) Residual
StdDev:      9.408521 7.162899

Variance function:
 Structure: Different standard deviations per stratum
 Formula: ~1 | leaf.type
 Parameter estimates:
        Grass W          Grass E      M. spinosum S. bracteolactus
      1.0000000        0.8851764        3.1796104        2.9647391
Fixed effects: list(A + w ~ leaf.type, m + s ~ 1)
                                Value Std.Error  DF   t-value p-value
A.(Intercept)                54.25350  5.988181 226  9.060097   0e+00
A.leaf.typeGrass E           30.92293  8.835210 226  3.499966   6e-04
A.leaf.typeM. spinosum      223.24504 15.915557 226 14.026844   0e+00
A.leaf.typeS. bracteolactus 240.27654 16.857356 226 14.253513   0e+00
w.(Intercept)                29.05581  1.712960 226 16.962334   0e+00
w.leaf.typeGrass E          -20.68938  2.592469 226 -7.980569   0e+00
w.leaf.typeM. spinosum       31.67297  7.753176 226  4.085161   1e-04
w.leaf.typeS. bracteolactus  26.97508  8.071111 226  3.342177   1e-03
m                            30.92881  2.065196 226 14.976213   0e+00
s                           -16.15406  2.365392 226 -6.829339   0e+00
 Correlation:
                            A.(In) A.l.GE A..M.s A..S.b w.(In) w.l.GE w..M.s w..S.b m
A.leaf.typeGrass E          -0.527
A.leaf.typeM. spinosum      -0.146  0.535
A.leaf.typeS. bracteolactus -0.115  0.537  0.746
w.(Intercept)               -0.217 -0.034 -0.200 -0.216
w.leaf.typeGrass E          -0.035 -0.283 -0.382 -0.399 -0.258
w.leaf.typeM. spinosum      -0.118 -0.227 -0.547 -0.454  0.157  0.573
w.leaf.typeS. bracteolactus -0.129 -0.241 -0.462 -0.575  0.188  0.601  0.640
m                           -0.217 -0.324 -0.633 -0.666  0.092  0.145  0.161  0.172
s                           -0.246 -0.354 -0.710 -0.748  0.416  0.575  0.702  0.752  0.544

Standardized Within-Group Residuals:
        Min          Q1         Med          Q3         Max
-2.27999668 -0.59657156 -0.02042043  0.57273326  3.12017985

Number of Observations: 247
Number of Groups: 12
```

This output is a result of the modelling process, which suggests m_1, m_2, m_3, s_1, s_1, s_2, s_3, $\sigma^2_{w_0}$, $\sigma^2_{m_0}$, $\sigma^2_{s_0}$ to be zero.

(3) Table of results (Table 2 in manuscript)

Parameter	Estimate	Meaning
μ_{A_0}	54.3% [42.5 : 66.0]	Maximum LFMC ($\hat{A}$) of grasses in the W site. A varies with plot and therefore a hyperparameter is estimated.
A_1	30.9% [13.5 : 48.3]	Difference between $\hat{A}$ of grasses in the E site and $\hat{A}$ of grasses in W site.
A_2	223.4% [191.9 : 254.6]	Difference between $\hat{A}$ of *M. spinosum* and $\hat{A}$ of grasses in the W site.
A_3	240.3% [207.1 : 273.5]	Difference between $\hat{A}$ of *S. filaginoides* and $\hat{A}$ of grasses in the W site.
w_0	29.1% [25.7 : 32.4]	Minimum LFMC ($\hat{w}$) of grasses in the W site.
w_1	-20.7% [-25.8 : -15.6]	Difference between $\hat{w}$ of grasses in the E site and $\hat{w}$ of grasses in the W site.
w_2	31.7% [16.4 : 47.0]	Difference between $\hat{w}$ of *M. spinosum* and $\hat{w}$ of grasses in the W site.
w_3	26.7% [11.1 : 42.9]	Difference between $\hat{w}$ of *S. filaginoides* and $\hat{w}$ of grasses in the W site.
m	30.9 days [26.9 : 35.0]	Day when the LFMC half-maximum occurs ($\hat{m}$) in the study area (data suggest one general value for all the leaf types).
s	-16.1 [-20.8 : -11.5]	Parameter controlling speed of change ($\hat{s}$) of the LFMC (data suggest one general value for all the leaf types, the negative value indicates vegetation to be drying during the fire season).
σ_{base}	7.1% [6.0 : 8.6]	LFMC variability within-plots (residual standard error) of grass ($\hat{\sigma}_{gw}$) in Site W at the beginning the fire season.
δ_1	0.9 [0.7 : 1.2]	Ratio between the within-plots standard error of the LFMC of grasses in Site E ($\hat{\sigma}_{ge}$) and grasses in W site ($\hat{\sigma}_{gw}$).
δ_2	3.2 [2.4 : 4.0]	Ratio between the within-plots standard error of the initial LFMC *M. spinosum* ($\hat{\sigma}_{sm}$) and grasses in W site ($\hat{\sigma}_{gw}$).
δ_3	3.0 [2.4 : 3.9]	Ratio between the within-plots standard error of the initial LFMC *S. filaginoides* ($\hat{\sigma}_{ss}$) and grasses in W site ($\hat{\sigma}_{gw}$)
σ_{A_0}	9.4% [3.9 : 15.8]	Variability among plots (standard deviation) in the maximum LFMC (random effects on *A*).

(4) Connection between model/table in manuscript and R output

**Parameter**	**R output**
μ_{A_0}	Fixed effects: list(A + w ~ leaf.type, m + s ~ 1)<br>Value Std.Error DF t-value p-value<br>A.(Intercept) 54.25350 5.988181 226 9.060097 0e+00
A_1	A.leaf.typeGrass E 30.92293 8.835210 226 3.499966 6e-04
A_2	A.leaf.typeM. spinosum 223.24504 15.915557 226 14.026844 0e+00
A_3	A.leaf.typeS. bracteolactus 240.27654 16.857356 226 14.253513 0e+00
w_0	w.(Intercept) 29.05581 1.712960 226 16.962334 0e+00
w_1	w.leaf.typeGrass E -20.68938 2.592469 226 -7.980569 0e+00
w_2	w.leaf.typeM. spinosum 31.67297 7.753176 226 4.085161 1e-04
w_3	w.leaf.typeS. bracteolactus 26.97508 8.071111 226 3.342177 1e-03
m	m 30.92881 2.065196 226 14.976213 0e+00
s	s -16.15406 2.365392 226 -6.829339 0e+00
σ_{base}	Random effects:<br>Formula: A ~ 1 | group<br>Residual<br>StdDev: 7.162899
δ_1	Variance function:<br>Structure: Different standard deviations per stratum<br>Formula: ~1 | leaf.type<br>Parameter estimates:<br>Grass E<br>0.8851764
δ_2	Variance function:<br>Structure: Different standard deviations per stratum<br>Formula: ~1 | leaf.type<br>Parameter estimates:<br>M. spinosum<br>3.1796104
δ_3	1.0000000 Variance function:<br>Structure: Different standard deviations per stratum<br>Formula: ~1 | leaf.type<br>Parameter estimates:<br>S. bracteolactus<br>2.9647391
σ_{A_0}	Random effects:<br>Formula: A ~ 1 | group<br>A.(Intercept)<br>StdDev: 9.408521
