# Supplementary material for: A nonlinear mixed‐effects modeling approach for ecological data: Using temporal dynamics of vegetation moisture as an example
Source: Ecol Evol. 2019 Aug 15;9(18):10225–40. doi: 10.1002/ece3.5543 (PMC6787861; doi:10.1002/ece3.5543)
Supplement: Supplementary file 5 [file ECE3-9-10225-s005.pdf]

Table 3. Live fuel moisture content (LFMC) average curves and LFMC curves per plot for each vegetation level (species/growth form) overall and plot level prediction for each leaf type. Average curves (fixed effect) and curves per plot (random effect) predict the LFMC as a function of the number of days since the first measurement (*t*) and are derived from fitted nonlinear mixed-effects model (Table 2).

Leaf type	Overall prediction	Plot level prediction
Grasses in the W site	$\text{LFMC} = \frac{25.2}{1 + e^{(30.9-t)/-16.1}} + 29.1$	$\text{LFMC}_{(plot\ 1)} = \frac{23.3}{1 + e^{(30.9-t)/-16.1}} + 29.1$ $\text{LFMC}_{(plot\ 2)} = \frac{23.3}{1 + e^{(30.9-t)/-16.1}} + 29.1$ $\text{LFMC}_{(plot\ 3)} = \frac{29.0}{1 + e^{(30.9-t)/-16.1}} + 29.1$
Grasses in the E site	$\text{LFMC} = \frac{76.8}{1 + e^{(30.9-t)/-16.1}} + 8.4$	$\text{LFMC}_{(plot\ 1)} = \frac{92.5}{1 + e^{(30.9-t)/-16.1}} + 8.4$ $\text{LFMC}_{(plot\ 2)} = \frac{65.7}{1 + e^{(30.9-t)/-16.1}} + 8.4$ $\text{LFMC}_{(plot\ 3)} = \frac{72.2}{1 + e^{(30.9-t)/-16.1}} + 8.4$
Mullinum spinosum	$\text{LFMC} = \frac{216.8}{1 + e^{(30.9-t)/-16.1}} + 60.7$	$\text{LFMC}_{(plot\ 1)} = \frac{223.7}{1 + e^{(30.9-t)/-16.1}} + 60.7$ $\text{LFMC}_{(plot\ 2)} = \frac{211.4}{1 + e^{(30.9-t)/-16.1}} + 60.7$ $\text{LFMC}_{(plot\ 3)} = \frac{215.2}{1 + e^{(30.9-t)/-16.1}} + 60.7$
S. filaginoides	$\text{LFMC} = \frac{238.5}{1 + e^{(30.9-t)/-16.1}} + 56.0$	$\text{LFMC}_{(plot\ 1)} = \frac{247.7}{1 + e^{(30.9-t)/-16.1}} + 56.0$ $\text{LFMC}_{(plot\ 2)} = \frac{240.9}{1 + e^{(30.9-t)/-16.1}} + 56.0$ $\text{LFMC}_{(plot\ 3)} = \frac{226.9}{1 + e^{(30.9-t)/-16.1}} + 56.0$
